# 4,4′,4′′-Tris(2-pyrid­yl)-2,2′,2′′-[(2,4,6-trimethyl­benzene-1,3,5-tri­yl)tris­(methyl­ene)tris­(sulfanedi­yl)]tripyrimidine

**DOI:** 10.1107/S1600536809026403

**Published:** 2009-07-11

**Authors:** Ya-Wen Zhang, Jian-Quan Wang, Lin Cheng

**Affiliations:** a School of Chemistry and Chemical Engineering, Southeast University, Nanjing 211189, People’s Republic of China

## Abstract

The title compound, C_39_H_33_N_9_S_3_, features a mesitylene unit substituted with three thio­ether arms. The distances from the center of mesitylene unit to the N atoms of the three pyridine rings in the arms are 10.05 (1), 9.94 (3) and 8.79 (3) Å. The crystal structure shows weak intra­molecular C—H⋯N hydrogen bonds.

## Related literature

For the potential use of tripodal ligands in the construction of organic-inorganic architectures, see: Hammes *et al.* (1998 ▶); Hiraoka *et al.* (2005 ▶). For the use of flexible thio­ether ligands to produce extended structures with metal ions, see: Dong *et al.* (2008*a* ▶,*b* ▶); Zhang *et al.* (2008 ▶).
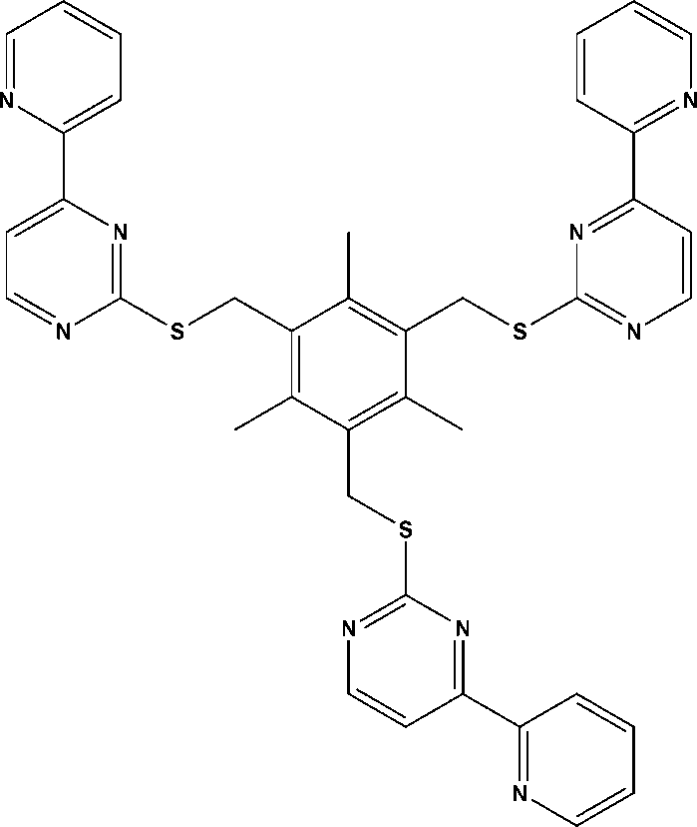


## Experimental

### 

#### Crystal data

C_39_H_33_N_9_S_3_
*M* *_r_* = 723.92
Monoclinic, 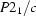

*a* = 11.966 (2) Å
*b* = 10.520 (2) Å
*c* = 31.959 (6) Å
β = 108.369 (6)°
*V* = 3818.1 (12) Å^3^
*Z* = 4
Mo *K*α radiation
μ = 0.24 mm^−1^
*T* = 293 K
0.25 × 0.20 × 0.18 mm

#### Data collection

Bruker SMART APEX CCD diffractometer
Absorption correction: multi-scan (*SADABS*; Sheldrick, 2000 ▶) *T*_min_ = 0.944, *T*_max_ = 0.959
17715 measured reflections
6544 independent reflections
2957 reflections with *I* > 2σ(*I*)
*R*_int_ = 0.107

#### Refinement

*R*[*F* ^2^ > 2σ(*F* ^2^)] = 0.081
*wR*(*F* ^2^) = 0.163
*S* = 1.08
6544 reflections
460 parameters
H-atom parameters constrained
Δρ_max_ = 0.89 e Å^−3^
Δρ_min_ = −0.26 e Å^−3^

### 

Data collection: *SMART* (Bruker, 2000 ▶); cell refinement: *SAINT* (Bruker, 2000 ▶); data reduction: *SAINT*; program(s) used to solve structure: *SHELXS97* (Sheldrick, 2008 ▶); program(s) used to refine structure: *SHELXL97* (Sheldrick, 2008 ▶); molecular graphics: *SHELXTL* (Sheldrick, 2008 ▶); software used to prepare material for publication: *SHELXTL*.

## Supplementary Material

Crystal structure: contains datablocks I, global. DOI: 10.1107/S1600536809026403/bt2991sup1.cif

Structure factors: contains datablocks I. DOI: 10.1107/S1600536809026403/bt2991Isup2.hkl

Additional supplementary materials:  crystallographic information; 3D view; checkCIF report

## Figures and Tables

**Table 1:** Hydrogen-bond geometry (Å, °)

*D*—H⋯*A*	*D*—H	H⋯*A*	*D*⋯*A*	*D*—H⋯*A*
C20—H20*A*⋯N5	0.97	2.39	2.818 (6)	106
C26—H26*A*⋯N5	0.93	2.45	2.767 (7)	100
C36—H36*A*⋯N7	0.93	2.49	2.806 (7)	100

**Fig. 1.**
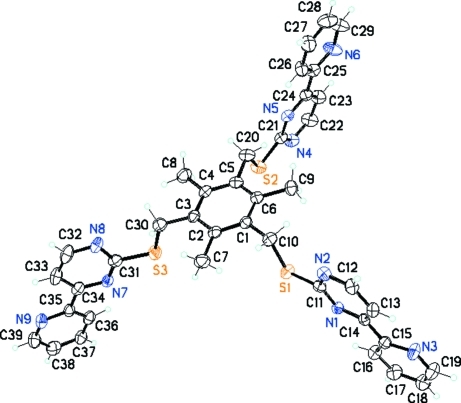
Structure of the title compound with 15% displacement ellipsoids.

**Fig. 2.**
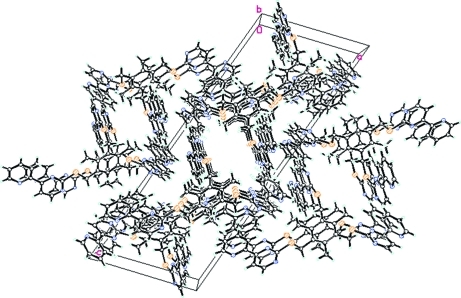
The three-dimensional supramolecular network of the title compound.

## supplementary crystallographic information

### Comment

Recent years have witnessed an explosion of great interest in tripodal ligands for their potential applications to construct intriguing hybrid organic-inorganic architectures and topologies (Hammes *et al.* 1998; Hiraoka *et al.*, 2005). On the other hand, flexible thioether ligands have been successfully used to produce various extended structures with metal ions (Dong *et al.*, 2008*a*,*b*; Zhang *et al.*, 2008). Herein, we report the crystal structure  of 2,2',2''-(2,4,6-trimethylbenzene-1,3,5-triyl) tris(methylene)tris(sulfanediyl)tris(4-(pyridin-2-yl)pyrimidine).

The tripodal character of C_33_H_30_N_10_S_3_ arises from the three thioether arms surrounding a central mesitylene.   The distances of the center of mesitylene to the nitrogen atoms of three pyridine rings in the arms are 10.05 (1), 9.94 (3) and 8.79 (3) °, respectively. The crystal structure shows wear intramolecular weak C—H···N hydrogen bonds.

### Experimental

An 95% ethanol solution (50 ml) of 1,3,5-tris(chloromethyl)-2,4,6-trimethylbenzene (2.64 g, 10 mmol) was added to a dry ethanol solution (300 ml) containing 4-(pyridin-2-yl)pyrimidine-2-thiol (5.67 g, 30 mmol) and sodium hydroxide (1.20 g, 30 mmol). The solution was stirred and refluxed for 8 h. Yellow precipitates were filtered out, washed by water and ethanol, and dried in vacuum. Yield (4.42 g) 61.0%. The yellow crystals were obtained after the filter slowly evaporated.

### Refinement

All the H atoms were located in a difference map and refined using a riding model with C-H ranging from 0.93Å to 0.97Å and *U*_iso_(H) = 1.2*U*_eq_(C) or *U*_iso_(H) = 1.5*U*_eq_(C_methyl_). There are holes in the structure but   the largest residual peak value is 0.892e/Å^3^, and no model for any solvent could be found.

### Crystal data

**Table:**

C_39_H_33_N_9_S_3_	*F*(000) = 1512
*M**_r_* = 723.92	*D*_x_ = 1.259 Mg m^−^^3^
Monoclinic, *P*2_1_/*c*	Mo *K*α radiation, λ = 0.71073 Å
Hall symbol: -P 2ybc	Cell parameters from 780 reflections
*a* = 11.966 (2) Å	θ = 2.4–28.0°
*b* = 10.520 (2) Å	µ = 0.24 mm^−^^1^
*c* = 31.959 (6) Å	*T* = 293 K
β = 108.369 (6)°	Block, yellow
*V* = 3818.1 (12) Å^3^	0.25 × 0.20 × 0.18 mm
*Z* = 4	

### Data collection

**Table:**

Bruker SMART APEX CCD diffractometer	6544 independent reflections
Radiation source: fine-focus sealed tube	2957 reflections with *I* > 2σ(*I*)
graphite	*R*_int_ = 0.107
φ and ω scans	θ_max_ = 25.1°, θ_min_ = 2.4°
Absorption correction: multi-scan (*SADABS*; Sheldrick, 2000)	*h* = −12→14
*T*_min_ = 0.944, *T*_max_ = 0.959	*k* = −12→11
17715 measured reflections	*l* = −38→38

### Refinement

**Table:**

Refinement on *F*^2^	Primary atom site location: structure-invariant direct methods
Least-squares matrix: full	Secondary atom site location: difference Fourier map
*R*[*F*^2^ > 2σ(*F*^2^)] = 0.081	Hydrogen site location: inferred from neighbouring sites
*wR*(*F*^2^) = 0.163	H-atom parameters constrained
*S* = 1.08	*w* = 1/[σ^2^(*F*_o_^2^) + (0.01*P*)^2^ + 1.28*P*] where *P* = (*F*_o_^2^ + 2*F*_c_^2^)/3
6544 reflections	(Δ/σ)_max_ < 0.001
460 parameters	Δρ_max_ = 0.89 e Å^−^^3^
0 restraints	Δρ_min_ = −0.26 e Å^−^^3^

### Special details

**Table:**

Geometry. All e.s.d.'s (except the e.s.d. in the dihedral angle between two l.s. planes) are estimated using the full covariance matrix. The cell e.s.d.'s are taken into account individually in the estimation of e.s.d.'s in distances, angles and torsion angles; correlations between e.s.d.'s in cell parameters are only used when they are defined by crystal symmetry. An approximate (isotropic) treatment of cell e.s.d.'s is used for estimating e.s.d.'s involving l.s. planes.
Refinement. Refinement of *F*^2^ against ALL reflections. The weighted *R*-factor *wR* and goodness of fit *S* are based on *F*^2^, conventional *R*-factors *R* are based on *F*, with *F* set to zero for negative *F*^2^. The threshold expression of *F*^2^ > σ(*F*^2^) is used only for calculating *R*-factors(gt) *etc*. and is not relevant to the choice of reflections for refinement. *R*-factors based on *F*^2^ are statistically about twice as large as those based on *F*, and *R*- factors based on ALL data will be even larger.

### Fractional atomic coordinates and isotropic or equivalent isotropic displacement parameters (Å^2^)

**Table:**

	*x*	*y*	*z*	*U*_iso_*/*U*_eq_	
S1	0.58642 (11)	−0.78603 (12)	−0.22867 (4)	0.0754 (4)	
S2	0.62715 (13)	−1.06746 (13)	−0.05185 (4)	0.0883 (5)	
S3	0.23200 (12)	−0.66957 (13)	−0.12895 (5)	0.0924 (5)	
C1	0.4361 (4)	−0.9300 (4)	−0.20145 (14)	0.0620 (12)	
C2	0.3434 (4)	−0.8618 (5)	−0.19733 (16)	0.0680 (13)	
C3	0.3010 (4)	−0.8814 (5)	−0.16132 (17)	0.0679 (13)	
C4	0.3590 (5)	−0.9676 (5)	−0.12875 (16)	0.0705 (13)	
C5	0.4568 (5)	−1.0361 (4)	−0.13279 (16)	0.0674 (13)	
C6	0.4962 (4)	−1.0149 (4)	−0.16834 (16)	0.0626 (12)	
C7	0.2812 (5)	−0.7628 (5)	−0.23179 (17)	0.0993 (17)	
H7A	0.3176	−0.7599	−0.2546	0.149*	
H7B	0.2871	−0.6809	−0.2180	0.149*	
H7C	0.1997	−0.7855	−0.2444	0.149*	
C8	0.3170 (5)	−0.9901 (5)	−0.08946 (17)	0.1026 (18)	
H8A	0.2498	−0.9373	−0.0919	0.154*	
H8B	0.3789	−0.9693	−0.0629	0.154*	
H8C	0.2956	−1.0778	−0.0887	0.154*	
C9	0.6043 (4)	−1.0849 (5)	−0.17075 (16)	0.0833 (15)	
H9A	0.6216	−1.0599	−0.1969	0.125*	
H9B	0.5900	−1.1748	−0.1715	0.125*	
H9C	0.6699	−1.0645	−0.1453	0.125*	
C10	0.4793 (4)	−0.9128 (5)	−0.24122 (14)	0.0740 (14)	
H10A	0.4139	−0.8914	−0.2671	0.089*	
H10B	0.5149	−0.9908	−0.2471	0.089*	
C11	0.6446 (4)	−0.7921 (4)	−0.27308 (14)	0.0616 (12)	
C12	0.6554 (5)	−0.8711 (5)	−0.33626 (15)	0.0713 (13)	
H12A	0.6288	−0.9253	−0.3604	0.086*	
C13	0.7498 (4)	−0.7944 (5)	−0.33389 (14)	0.0645 (12)	
H13A	0.7859	−0.7955	−0.3558	0.077*	
C14	0.7884 (4)	−0.7169 (4)	−0.29843 (14)	0.0532 (11)	
C15	0.8931 (4)	−0.6337 (4)	−0.29058 (15)	0.0626 (12)	
C16	0.9256 (5)	−0.5489 (5)	−0.25625 (15)	0.0745 (14)	
H16A	0.8817	−0.5408	−0.2370	0.089*	
C17	1.0242 (5)	−0.4764 (5)	−0.2511 (2)	0.0908 (17)	
H17A	1.0490	−0.4194	−0.2277	0.109*	
C18	1.0852 (5)	−0.4882 (6)	−0.2800 (2)	0.0940 (17)	
H18A	1.1508	−0.4379	−0.2777	0.113*	
C19	1.0482 (6)	−0.5740 (6)	−0.3118 (2)	0.1053 (19)	
H19A	1.0927	−0.5843	−0.3308	0.126*	
C20	0.5140 (5)	−1.1352 (4)	−0.09856 (16)	0.0861 (16)	
H20A	0.5488	−1.2005	−0.1120	0.103*	
H20B	0.4543	−1.1750	−0.0883	0.103*	
C21	0.7111 (4)	−1.2020 (5)	−0.03108 (15)	0.0686 (13)	
C22	0.8719 (6)	−1.2798 (7)	0.02049 (17)	0.0975 (19)	
H22A	0.9365	−1.2690	0.0456	0.117*	
C23	0.8548 (5)	−1.3965 (6)	0.00115 (17)	0.0937 (17)	
H23A	0.9057	−1.4640	0.0123	0.112*	
C24	0.7563 (5)	−1.4094 (5)	−0.03647 (16)	0.0699 (13)	
C25	0.7259 (5)	−1.5296 (5)	−0.06142 (17)	0.0773 (14)	
C26	0.6307 (5)	−1.5380 (6)	−0.09824 (19)	0.0870 (16)	
H26A	0.5843	−1.4669	−0.1087	0.104*	
C27	0.6038 (6)	−1.6508 (8)	−0.1196 (2)	0.108 (2)	
H27A	0.5392	−1.6576	−0.1449	0.129*	
C28	0.6720 (9)	−1.7529 (7)	−0.1036 (2)	0.120 (2)	
H28A	0.6549	−1.8316	−0.1175	0.144*	
C29	0.7660 (9)	−1.7382 (7)	−0.0670 (3)	0.140 (3)	
H29A	0.8116	−1.8098	−0.0562	0.168*	
C30	0.1921 (5)	−0.8156 (5)	−0.15912 (17)	0.0876 (15)	
H30A	0.1501	−0.8700	−0.1446	0.105*	
H30B	0.1406	−0.7979	−0.1887	0.105*	
C31	0.0955 (5)	−0.6028 (6)	−0.13059 (16)	0.0783 (15)	
C32	−0.0991 (6)	−0.6111 (7)	−0.15071 (19)	0.105 (2)	
H32A	−0.1702	−0.6522	−0.1643	0.126*	
C33	−0.1041 (5)	−0.4911 (7)	−0.13345 (17)	0.0944 (18)	
H33A	−0.1750	−0.4513	−0.1356	0.113*	
C34	0.0034 (5)	−0.4348 (6)	−0.11291 (15)	0.0763 (15)	
C35	0.0108 (5)	−0.3091 (5)	−0.09093 (16)	0.0720 (13)	
C36	0.1165 (5)	−0.2639 (6)	−0.06459 (16)	0.0807 (15)	
H36A	0.1845	−0.3123	−0.0598	0.097*	
C37	0.1220 (6)	−0.1456 (7)	−0.04502 (18)	0.0881 (16)	
H37A	0.1930	−0.1120	−0.0273	0.106*	
C38	0.0216 (8)	−0.0821 (6)	−0.0527 (2)	0.1056 (19)	
H38A	0.0216	−0.0022	−0.0403	0.127*	
C39	−0.0823 (7)	−0.1338 (7)	−0.0790 (2)	0.1069 (19)	
H39A	−0.1514	−0.0877	−0.0834	0.128*	
N1	0.7367 (3)	−0.7132 (3)	−0.26700 (11)	0.0583 (9)	
N2	0.5992 (3)	−0.8731 (3)	−0.30604 (13)	0.0677 (10)	
N3	0.9526 (4)	−0.6468 (4)	−0.31899 (14)	0.0890 (13)	
N4	0.8019 (4)	−1.1798 (4)	0.00566 (14)	0.0881 (13)	
N5	0.6858 (3)	−1.3116 (4)	−0.05227 (11)	0.0648 (10)	
N6	0.7987 (5)	−1.6291 (5)	−0.04492 (15)	0.1137 (17)	
N7	0.1042 (4)	−0.4918 (4)	−0.11146 (12)	0.0690 (11)	
N8	−0.0016 (4)	−0.6716 (4)	−0.14938 (13)	0.0852 (13)	
N9	−0.0891 (4)	−0.2464 (5)	−0.09860 (15)	0.0893 (13)	

### Atomic displacement parameters (Å^2^)

**Table:**

	*U*^11^	*U*^22^	*U*^33^	*U*^12^	*U*^13^	*U*^23^
S1	0.0783 (9)	0.0869 (10)	0.0638 (8)	−0.0206 (7)	0.0263 (7)	−0.0125 (7)
S2	0.0980 (10)	0.0786 (10)	0.0728 (9)	−0.0051 (8)	0.0046 (7)	−0.0037 (7)
S3	0.0623 (9)	0.0896 (11)	0.1195 (12)	−0.0098 (8)	0.0202 (8)	−0.0139 (9)
C1	0.053 (3)	0.069 (3)	0.060 (3)	−0.010 (3)	0.012 (3)	−0.002 (3)
C2	0.052 (3)	0.071 (3)	0.069 (3)	−0.015 (3)	0.002 (3)	0.005 (3)
C3	0.052 (3)	0.072 (3)	0.077 (3)	−0.010 (3)	0.017 (3)	0.004 (3)
C4	0.069 (3)	0.079 (4)	0.066 (3)	−0.019 (3)	0.025 (3)	0.001 (3)
C5	0.067 (3)	0.061 (3)	0.060 (3)	−0.011 (3)	−0.001 (3)	−0.001 (2)
C6	0.058 (3)	0.067 (3)	0.055 (3)	−0.013 (3)	0.007 (3)	−0.006 (3)
C7	0.096 (4)	0.097 (4)	0.097 (4)	0.001 (3)	0.019 (3)	0.030 (3)
C8	0.105 (4)	0.119 (5)	0.096 (4)	−0.006 (4)	0.048 (4)	0.022 (3)
C9	0.070 (3)	0.096 (4)	0.079 (3)	0.002 (3)	0.018 (3)	−0.009 (3)
C10	0.074 (3)	0.084 (4)	0.060 (3)	−0.018 (3)	0.015 (3)	−0.005 (3)
C11	0.061 (3)	0.062 (3)	0.054 (3)	0.008 (3)	0.008 (2)	0.008 (2)
C12	0.072 (4)	0.084 (4)	0.056 (3)	0.004 (3)	0.017 (3)	−0.016 (3)
C13	0.054 (3)	0.085 (4)	0.051 (3)	0.002 (3)	0.011 (2)	−0.005 (3)
C14	0.050 (3)	0.058 (3)	0.048 (3)	0.008 (2)	0.011 (2)	0.002 (2)
C15	0.056 (3)	0.070 (3)	0.057 (3)	0.004 (3)	0.009 (3)	0.006 (3)
C16	0.071 (4)	0.089 (4)	0.059 (3)	−0.011 (3)	0.014 (3)	−0.008 (3)
C17	0.080 (4)	0.092 (4)	0.087 (4)	−0.021 (3)	0.008 (4)	−0.013 (3)
C18	0.066 (4)	0.106 (5)	0.101 (5)	−0.018 (3)	0.012 (4)	−0.010 (4)
C19	0.085 (5)	0.132 (5)	0.110 (5)	−0.024 (4)	0.046 (4)	−0.010 (4)
C20	0.088 (4)	0.072 (3)	0.076 (3)	−0.017 (3)	−0.007 (3)	0.003 (3)
C21	0.066 (3)	0.080 (4)	0.054 (3)	−0.015 (3)	0.011 (3)	−0.001 (3)
C22	0.109 (5)	0.090 (5)	0.060 (3)	−0.007 (4)	−0.020 (3)	−0.003 (4)
C23	0.105 (5)	0.096 (5)	0.066 (4)	0.005 (4)	0.007 (3)	0.012 (3)
C24	0.080 (4)	0.073 (4)	0.054 (3)	−0.006 (3)	0.016 (3)	0.010 (3)
C25	0.081 (4)	0.083 (4)	0.063 (3)	−0.006 (3)	0.016 (3)	0.004 (3)
C26	0.072 (4)	0.096 (5)	0.088 (4)	0.000 (3)	0.018 (3)	−0.024 (3)
C27	0.093 (5)	0.115 (6)	0.111 (5)	−0.007 (5)	0.027 (4)	−0.029 (5)
C28	0.169 (7)	0.094 (6)	0.091 (5)	−0.047 (5)	0.031 (5)	−0.023 (4)
C29	0.222 (9)	0.072 (5)	0.104 (5)	0.009 (5)	0.021 (6)	0.000 (4)
C30	0.080 (4)	0.078 (4)	0.103 (4)	−0.013 (3)	0.026 (3)	−0.010 (3)
C31	0.080 (4)	0.087 (4)	0.059 (3)	−0.010 (3)	0.009 (3)	0.014 (3)
C32	0.057 (4)	0.162 (7)	0.086 (4)	−0.028 (4)	0.009 (3)	−0.022 (4)
C33	0.067 (4)	0.146 (6)	0.078 (4)	−0.009 (4)	0.034 (3)	−0.021 (4)
C34	0.063 (4)	0.104 (5)	0.056 (3)	−0.015 (4)	0.011 (3)	0.014 (3)
C35	0.068 (4)	0.090 (4)	0.066 (3)	0.004 (3)	0.032 (3)	0.007 (3)
C36	0.069 (4)	0.107 (5)	0.068 (3)	−0.007 (3)	0.024 (3)	0.003 (3)
C37	0.079 (4)	0.103 (5)	0.090 (4)	−0.006 (4)	0.039 (3)	−0.008 (4)
C38	0.130 (6)	0.111 (5)	0.089 (4)	−0.001 (5)	0.053 (5)	−0.006 (4)
C39	0.093 (5)	0.121 (6)	0.119 (5)	0.009 (4)	0.050 (4)	−0.010 (5)
N1	0.052 (2)	0.058 (2)	0.060 (2)	−0.004 (2)	0.0120 (19)	−0.0016 (19)
N2	0.066 (3)	0.068 (3)	0.060 (2)	−0.004 (2)	0.007 (2)	−0.014 (2)
N3	0.070 (3)	0.115 (4)	0.093 (3)	−0.023 (3)	0.042 (3)	−0.023 (3)
N4	0.104 (4)	0.083 (3)	0.067 (3)	−0.014 (3)	0.013 (3)	−0.007 (2)
N5	0.065 (3)	0.074 (3)	0.052 (2)	−0.013 (2)	0.0129 (19)	0.002 (2)
N6	0.162 (5)	0.076 (3)	0.079 (3)	0.017 (4)	0.003 (3)	0.007 (3)
N7	0.055 (3)	0.080 (3)	0.073 (3)	−0.004 (2)	0.021 (2)	0.003 (2)
N8	0.059 (3)	0.119 (4)	0.080 (3)	−0.023 (3)	0.024 (2)	−0.009 (3)
N9	0.074 (3)	0.108 (4)	0.093 (3)	0.005 (3)	0.036 (3)	−0.003 (3)

### Geometric parameters (Å, °)

**Table:**

S1—C11	1.768 (5)	C18—H18A	0.9300
S1—C10	1.805 (5)	C19—N3	1.336 (7)
S2—C21	1.741 (5)	C19—H19A	0.9300
S2—C20	1.815 (4)	C20—H20A	0.9700
S3—C31	1.763 (6)	C20—H20B	0.9700
S3—C30	1.796 (5)	C21—N5	1.323 (5)
C1—C2	1.361 (6)	C21—N4	1.345 (6)
C1—C6	1.398 (6)	C22—N4	1.335 (6)
C1—C10	1.527 (6)	C22—C23	1.361 (6)
C2—C3	1.412 (6)	C22—H22A	0.9300
C2—C7	1.528 (6)	C23—C24	1.400 (7)
C3—C4	1.390 (6)	C23—H23A	0.9300
C3—C30	1.497 (7)	C24—N5	1.324 (6)
C4—C5	1.415 (7)	C24—C25	1.478 (7)
C4—C8	1.511 (7)	C25—N6	1.357 (6)
C5—C6	1.379 (7)	C25—C26	1.358 (7)
C5—C20	1.510 (6)	C26—C27	1.355 (7)
C6—C9	1.511 (7)	C26—H26A	0.9300
C7—H7A	0.9600	C27—C28	1.349 (8)
C7—H7B	0.9600	C27—H27A	0.9300
C7—H7C	0.9600	C28—C29	1.352 (9)
C8—H8A	0.9600	C28—H28A	0.9300
C8—H8B	0.9600	C29—N6	1.340 (7)
C8—H8C	0.9600	C29—H29A	0.9300
C9—H9A	0.9600	C30—H30A	0.9700
C9—H9B	0.9600	C30—H30B	0.9700
C9—H9C	0.9600	C31—N7	1.307 (6)
C10—H10A	0.9700	C31—N8	1.339 (6)
C10—H10B	0.9700	C32—N8	1.319 (7)
C11—N2	1.330 (5)	C32—C33	1.386 (7)
C11—N1	1.344 (5)	C32—H32A	0.9300
C12—N2	1.340 (6)	C33—C34	1.380 (7)
C12—C13	1.371 (6)	C33—H33A	0.9300
C12—H12A	0.9300	C34—N7	1.334 (6)
C13—C14	1.353 (5)	C34—C35	1.487 (7)
C13—H13A	0.9300	C35—N9	1.319 (6)
C14—N1	1.336 (5)	C35—C36	1.365 (7)
C14—C15	1.484 (6)	C36—C37	1.386 (7)
C15—N3	1.325 (6)	C36—H36A	0.9300
C15—C16	1.371 (6)	C37—C38	1.328 (8)
C16—C17	1.370 (7)	C37—H37A	0.9300
C16—H16A	0.9300	C38—C39	1.375 (8)
C17—C18	1.352 (8)	C38—H38A	0.9300
C17—H17A	0.9300	C39—N9	1.329 (7)
C18—C19	1.328 (7)	C39—H39A	0.9300
			
C11—S1—C10	103.1 (2)	S2—C20—H20A	109.2
C21—S2—C20	100.7 (2)	C5—C20—H20B	109.2
C31—S3—C30	103.7 (3)	S2—C20—H20B	109.2
C2—C1—C6	120.3 (4)	H20A—C20—H20B	107.9
C2—C1—C10	121.2 (4)	N5—C21—N4	126.1 (5)
C6—C1—C10	118.5 (5)	N5—C21—S2	120.1 (4)
C1—C2—C3	121.0 (4)	N4—C21—S2	113.7 (4)
C1—C2—C7	121.1 (5)	N4—C22—C23	124.2 (5)
C3—C2—C7	117.9 (5)	N4—C22—H22A	117.9
C4—C3—C2	118.9 (5)	C23—C22—H22A	117.9
C4—C3—C30	120.2 (5)	C22—C23—C24	116.3 (5)
C2—C3—C30	120.8 (5)	C22—C23—H23A	121.9
C3—C4—C5	119.7 (4)	C24—C23—H23A	121.9
C3—C4—C8	120.4 (5)	N5—C24—C23	121.0 (5)
C5—C4—C8	119.9 (5)	N5—C24—C25	116.1 (5)
C6—C5—C4	120.0 (4)	C23—C24—C25	122.9 (5)
C6—C5—C20	120.9 (5)	N6—C25—C26	123.1 (5)
C4—C5—C20	119.0 (5)	N6—C25—C24	115.6 (5)
C5—C6—C1	119.9 (5)	C26—C25—C24	121.3 (5)
C5—C6—C9	118.9 (4)	C27—C26—C25	119.7 (6)
C1—C6—C9	121.2 (4)	C27—C26—H26A	120.2
C2—C7—H7A	109.5	C25—C26—H26A	120.2
C2—C7—H7B	109.5	C28—C27—C26	119.2 (6)
H7A—C7—H7B	109.5	C28—C27—H27A	120.4
C2—C7—H7C	109.5	C26—C27—H27A	120.4
H7A—C7—H7C	109.5	C27—C28—C29	118.3 (7)
H7B—C7—H7C	109.5	C27—C28—H28A	120.8
C4—C8—H8A	109.5	C29—C28—H28A	120.8
C4—C8—H8B	109.5	N6—C29—C28	125.4 (7)
H8A—C8—H8B	109.5	N6—C29—H29A	117.3
C4—C8—H8C	109.5	C28—C29—H29A	117.3
H8A—C8—H8C	109.5	C3—C30—S3	109.4 (3)
H8B—C8—H8C	109.5	C3—C30—H30A	109.8
C6—C9—H9A	109.5	S3—C30—H30A	109.8
C6—C9—H9B	109.5	C3—C30—H30B	109.8
H9A—C9—H9B	109.5	S3—C30—H30B	109.8
C6—C9—H9C	109.5	H30A—C30—H30B	108.2
H9A—C9—H9C	109.5	N7—C31—N8	128.5 (5)
H9B—C9—H9C	109.5	N7—C31—S3	113.9 (4)
C1—C10—S1	107.8 (3)	N8—C31—S3	117.5 (5)
C1—C10—H10A	110.1	N8—C32—C33	125.1 (5)
S1—C10—H10A	110.1	N8—C32—H32A	117.4
C1—C10—H10B	110.1	C33—C32—H32A	117.4
S1—C10—H10B	110.1	C34—C33—C32	115.4 (6)
H10A—C10—H10B	108.5	C34—C33—H33A	122.3
N2—C11—N1	128.4 (4)	C32—C33—H33A	122.3
N2—C11—S1	119.6 (4)	N7—C34—C33	121.3 (6)
N1—C11—S1	111.9 (3)	N7—C34—C35	117.7 (5)
N2—C12—C13	124.1 (4)	C33—C34—C35	120.9 (6)
N2—C12—H12A	117.9	N9—C35—C36	123.3 (5)
C13—C12—H12A	117.9	N9—C35—C34	116.2 (5)
C14—C13—C12	117.3 (4)	C36—C35—C34	120.4 (6)
C14—C13—H13A	121.4	C35—C36—C37	119.6 (6)
C12—C13—H13A	121.4	C35—C36—H36A	120.2
N1—C14—C13	122.1 (4)	C37—C36—H36A	120.2
N1—C14—C15	115.0 (4)	C38—C37—C36	117.2 (6)
C13—C14—C15	122.9 (4)	C38—C37—H37A	121.4
N3—C15—C16	122.6 (5)	C36—C37—H37A	121.4
N3—C15—C14	115.4 (4)	C37—C38—C39	120.4 (6)
C16—C15—C14	122.0 (5)	C37—C38—H38A	119.8
C17—C16—C15	118.3 (5)	C39—C38—H38A	119.8
C17—C16—H16A	120.9	N9—C39—C38	123.3 (6)
C15—C16—H16A	120.9	N9—C39—H39A	118.3
C18—C17—C16	119.8 (5)	C38—C39—H39A	118.4
C18—C17—H17A	120.1	C14—N1—C11	115.2 (4)
C16—C17—H17A	120.1	C11—N2—C12	112.9 (4)
C19—C18—C17	117.7 (6)	C15—N3—C19	116.0 (5)
C19—C18—H18A	121.1	C22—N4—C21	114.6 (4)
C17—C18—H18A	121.1	C21—N5—C24	117.8 (4)
C18—C19—N3	125.5 (6)	C29—N6—C25	114.2 (5)
C18—C19—H19A	117.2	C31—N7—C34	116.6 (5)
N3—C19—H19A	117.2	C32—N8—C31	112.8 (5)
C5—C20—S2	112.0 (3)	C35—N9—C39	116.2 (5)
C5—C20—H20A	109.2		
			
C6—C1—C2—C3	−4.4 (7)	N6—C25—C26—C27	1.4 (8)
C10—C1—C2—C3	177.6 (4)	C24—C25—C26—C27	−178.2 (5)
C6—C1—C2—C7	176.3 (4)	C25—C26—C27—C28	0.4 (9)
C10—C1—C2—C7	−1.6 (6)	C26—C27—C28—C29	−0.8 (10)
C1—C2—C3—C4	3.1 (7)	C27—C28—C29—N6	−0.7 (12)
C7—C2—C3—C4	−177.7 (4)	C4—C3—C30—S3	90.2 (5)
C1—C2—C3—C30	−174.0 (4)	C2—C3—C30—S3	−92.7 (5)
C7—C2—C3—C30	5.2 (6)	C31—S3—C30—C3	178.6 (4)
C2—C3—C4—C5	−1.6 (6)	C30—S3—C31—N7	−177.8 (4)
C30—C3—C4—C5	175.5 (4)	C30—S3—C31—N8	4.4 (4)
C2—C3—C4—C8	179.3 (4)	N8—C32—C33—C34	−1.1 (8)
C30—C3—C4—C8	−3.6 (7)	C32—C33—C34—N7	2.2 (7)
C3—C4—C5—C6	1.6 (7)	C32—C33—C34—C35	−176.8 (4)
C8—C4—C5—C6	−179.3 (4)	N7—C34—C35—N9	169.9 (4)
C3—C4—C5—C20	−176.2 (4)	C33—C34—C35—N9	−11.1 (7)
C8—C4—C5—C20	2.9 (6)	N7—C34—C35—C36	−9.9 (6)
C4—C5—C6—C1	−2.9 (6)	C33—C34—C35—C36	169.1 (5)
C20—C5—C6—C1	174.9 (4)	N9—C35—C36—C37	−1.0 (7)
C4—C5—C6—C9	177.2 (4)	C34—C35—C36—C37	178.8 (4)
C20—C5—C6—C9	−4.9 (6)	C35—C36—C37—C38	0.9 (7)
C2—C1—C6—C5	4.4 (6)	C36—C37—C38—C39	0.1 (8)
C10—C1—C6—C5	−177.6 (4)	C37—C38—C39—N9	−1.2 (9)
C2—C1—C6—C9	−175.8 (4)	C13—C14—N1—C11	0.3 (6)
C10—C1—C6—C9	2.2 (6)	C15—C14—N1—C11	−177.5 (3)
C2—C1—C10—S1	88.1 (5)	N2—C11—N1—C14	0.3 (6)
C6—C1—C10—S1	−89.9 (4)	S1—C11—N1—C14	177.2 (3)
C11—S1—C10—C1	171.6 (3)	N1—C11—N2—C12	−0.5 (6)
C10—S1—C11—N2	4.4 (4)	S1—C11—N2—C12	−177.2 (3)
C10—S1—C11—N1	−172.8 (3)	C13—C12—N2—C11	0.0 (6)
N2—C12—C13—C14	0.6 (7)	C16—C15—N3—C19	1.3 (7)
C12—C13—C14—N1	−0.7 (6)	C14—C15—N3—C19	−178.9 (4)
C12—C13—C14—C15	176.9 (4)	C18—C19—N3—C15	−2.4 (9)
N1—C14—C15—N3	172.9 (4)	C23—C22—N4—C21	−0.7 (8)
C13—C14—C15—N3	−4.8 (6)	N5—C21—N4—C22	1.3 (7)
N1—C14—C15—C16	−7.2 (6)	S2—C21—N4—C22	−175.8 (4)
C13—C14—C15—C16	175.0 (4)	N4—C21—N5—C24	−0.6 (7)
N3—C15—C16—C17	−0.8 (7)	S2—C21—N5—C24	176.3 (3)
C14—C15—C16—C17	179.4 (4)	C23—C24—N5—C21	−0.6 (7)
C15—C16—C17—C18	1.2 (8)	C25—C24—N5—C21	−179.2 (4)
C16—C17—C18—C19	−2.2 (8)	C28—C29—N6—C25	2.3 (10)
C17—C18—C19—N3	2.9 (9)	C26—C25—N6—C29	−2.7 (8)
C6—C5—C20—S2	94.4 (5)	C24—C25—N6—C29	177.0 (5)
C4—C5—C20—S2	−87.7 (5)	N8—C31—N7—C34	−4.6 (7)
C21—S2—C20—C5	−157.7 (4)	S3—C31—N7—C34	177.9 (3)
C20—S2—C21—N5	4.7 (4)	C33—C34—N7—C31	0.3 (7)
C20—S2—C21—N4	−178.0 (4)	C35—C34—N7—C31	179.3 (4)
N4—C22—C23—C24	−0.4 (9)	C33—C32—N8—C31	−2.2 (8)
C22—C23—C24—N5	1.1 (7)	N7—C31—N8—C32	5.4 (7)
C22—C23—C24—C25	179.6 (5)	S3—C31—N8—C32	−177.2 (4)
N5—C24—C25—N6	180.0 (5)	C36—C35—N9—C39	0.1 (7)
C23—C24—C25—N6	1.5 (7)	C34—C35—N9—C39	−179.8 (4)
N5—C24—C25—C26	−0.3 (7)	C38—C39—N9—C35	1.0 (8)
C23—C24—C25—C26	−178.8 (5)		

### Hydrogen-bond geometry (Å, °)

**Table:**

*D*—H···*A*	*D*—H	H···*A*	*D*···*A*	*D*—H···*A*
C20—H20A···N5	0.97	2.39	2.818 (6)	106
C26—H26A···N5	0.93	2.45	2.767 (7)	100
C36—H36A···N7	0.93	2.49	2.806 (7)	100
